# Factors associated with 24-hour urinary volume: the Swiss salt survey

**DOI:** 10.1186/1471-2369-14-246

**Published:** 2013-11-07

**Authors:** Tobias Schoen, Jonas Blum, Fred Paccaud, Michel Burnier, Murielle Bochud, David Conen

**Affiliations:** 1 Department of Medicine, University Hospital, Basel, Switzerland; 2 Community Prevention Unit, Institute of Social and Preventive Medicine (IUMSP), Lausanne University Hospital, Lausanne, Switzerland; 3 Service of Nephrology and Hypertension, Lausanne University Hospital, Lausanne, Switzerland

**Keywords:** Daily urinary volume, Kidney function, Prevention, Population based study, Salt, Gender, Salt excretion

## Abstract

**Background:**

Low 24-hour urine volume (24UV) may be a significant risk factor for decline in kidney function. We therefore aimed to study associated markers and possible determinants of 24UV in a sample of the Swiss population.

**Methods:**

The cross-sectional Swiss Salt Study included a population-based sample of 1535 (746 men and 789 women) individuals from three linguistic regions of Switzerland. Data from 1300 subjects were available for the present analysis. 24UV was measured using 24-hour urine collection. Determinants of 24UV were identified using multivariable linear regression models.

**Results:**

In bivariate analysis, 24UV was higher in women compared to men (2000 ml/24 h [interquartile range (IQR): 1354, 2562] versus 1780 ml/24 h [IQR: 1244, 2360], p = 0.002). In multivariable regression analyses, independent associated markers of 24UV were female sex (β = 280, 95% confidence interval [CI]: 174, 386, p < 0.0001), fluid intake (β = 604, 95% CI: 539, 670, p < 0.0001), sodium excretion (β = 4.2, 95% CI: 3.4, 4.9, p < 0.0001) age (β = 6.6, CI: 3.4, 9.7, p < .0001), creatinine clearance (β = 2.4, CI: 0.2, 4.6, p = 0.04), living in the German-speaking part of Switzerland (β = 124, CI: 29, 219, p = 0.01), alcohol consumption (β = 41, CI: 9, 73, p = 0.01 for increasing categories of alcohol consumption), body mass index (β = −32, CI: -45, -18, p < 0.0001), current smoking (β = −146, CI: -265, -26, p = 0.02), and consumption of meat and cold cut (β = −56, CI: -108, -5, p = 0.03).

**Conclusion:**

In this large population-based, cross-sectional study, we found several strong and independent correlates for 24UV. These findings may be important to improve our understanding in the development of chronic kidney disease.

## Background

Chronic kidney disease (CKD) is a major public health burden worldwide, both as a disabling disease and as a risk factor for adverse outcomes [1]. Major determinants of CKD include age, hypertension and type 2 diabetes [2]. Several studies also found that low 24-hour urine volume (24UV) may be a significant risk factor for a decline in kidney function [3]. Participants of the prospective Walkerton Health Study showed an inverse, graded relationship between 24UV and the 6-year decline in estimated glomerular filtration rate (eGFR). In addition, recent studies raised the possibility that low 24UV increases the susceptibility to hypertension and urolithiasis [4-6]. Thus, 24UV may not only be a marker of renal function (and hence an inexpensive monitoring tool), but it may also be a significant step in the process heading to the development of hypertension, kidney failure and urolithiasis. However, few large population based studies have investigated factors associated with 24UV. Some of these studies found a lower 24UV in men compared to women [6], which may explain the male predominance in urolithiasis [4,5] or even with cardiovascular and renal diseases. Therefore, the aim of this study was to examine factors associated with 24UV in a population-based cross-sectional study.

## Methods

### Study participants

All study participants were drawn from the Swiss Salt Study (SSS), a random population-based cross-sectional sample of 1535 individuals from the three main linguistic regions of Switzerland. The SSS complied with the Declaration of Helsinki and was approved by the responsible local Institutional Ethics Committees. All participants gave written informed consent. For participants below age 18 years, written consent from one parent or a legal representative was obtained. Details of the conduct of this study have been previously published [7]. Of the original sample, 235 individuals were excluded due to missing information on fluid intake (n = 79), refusal of providing a blood sample (n = 144), self-reported missed portion of urine collection (n = 25) or 24-hour urine volume <500 ml (n = 12), leaving 1300 individuals for the present analysis.

### Study centers

Eleven centers in Switzerland were included representing the three main linguistic regions of the country: 4 German-speaking cantons (Basel, Lucerne, St Gallen and Zurich) and 5 non German-speaking cantons (Fribourg, Geneva, Vaud, Valais [all French-speaking] and Ticino [Italian-speaking]). Recruitment started in January 2010 and was completed in March 2012.

### Inclusion and exclusion criteria

To be included in the study, participants had to be ≥15 years old and permanent residents in Switzerland. People were excluded from the survey if they were living in institutions, had linguistic difficulties preventing them from understanding study objectives, instructions or questions, or if they were not able or not willing to collect a 24-hour urine sample.

### Sampling strategy

Each study center recruited participants among the population of the corresponding canton and aimed to include the same number of people in each of the eight predefined sex and age strata (15–29, 30–44, 45–59 and ≥60 years). A two-level sampling strategy was used to recruit participants. The Swiss Federal Statistical Office provided a list of randomly selected households from the Swisscom fixed line directory (first level), separately for each canton. The study centers sent an information letter to these households and contacted them by phone a few days later; for each household we made up to three phone call attempts on different days. One person per household was then randomly selected (second level) by using a computer generated random number and invited to participate in the study.

### Study measurements

The study consisted of two visits at the study center, before and after the 24-hour urine collection. At the first visit, weight and height were measured and the body mass index (BMI) was calculated as weight divided by height in square meter. At the second visit, participants were asked to fill in a questionnaire about lifestyle, medical history and salt-related health issues. Resting blood pressure was measured in the sitting position in a quiet environment using an appropriately sized cuff and the same validated oscillometric device in all centers (Omron HEM-907, Omron Health Care, Matsusaka, Japan). Hypertension was defined as mean blood pressure of at least 140/90 mmHg.

### Ascertainment of 24 h-urine volume

At the end of the first visit, participants were given two 3-liter plastic bottles and standardized instructions on how to collect urine [7]. They were asked to first empty their bladder, discard the urine and write down the time (start of the collection). The end of the collection was determined by the last urine void in the study center at the second visit or, if not possible, by the time of the last void. Participants were asked whether any collection of urine was lost or forgotten.

In the study center, the urine was mixed and weighed before aliquots were sampled. Urinary samples were then stored at −20°C in the study centers and subsequently sent to the core lab at the Centre Hospitalier Universitaire Vaudois (CHUV, Lausanne, Switzerland) for centralized analyses with standard methods and stringent internal quality controls. Serum creatinine (intra- and inter-batch coefficients of variation: 0.70% and 2.30%) and urine creatinine (2.10% and 2.20% for concentration around 1.10 mmol/L; 1.30% and 1.70% for concentrations around 1.80 mmol/L and 1.10% and 1.20% for concentrations around 5.30 mmol/L) were measured by Jaffé kinetic compensated method. The manufacturer was Roche Diagnostics, Switzerland. Creatinine clearance was estimated by using the following formula:

$$\left(U_{\text{creatinine}\left[\mu \text{mol}/L\right]}*V_{\left[\text{mL}/\text{min}\right]}\right)/\text{Seru}m_{\text{creatinine}\left[\mu \text{mol}/L\right]}.$$

### Statistical analysis

To describe unadjusted continuous variables we used median and interquartile range, categorical variables were displayed as percentages. Wilcoxon rank-sum tests (for continuous variables) and chi-squared test (for categorical variables) were used to compare group characteristics, as appropriate. Baseline characteristics were compared according to sex and 24UV defined as low and high according to the lowest and highest quartile of 24UV. To analyze factors associated with 24UV, multiple linear regression models were constructed with 24UV as the dependent variable. Age, sex, BMI, oral anti-diabetic medication, hypertension, creatinine clearance, and smoking categories were included as covariates in both models as well as linguistic region (German vs. non-German), level of education, season, dietary habits (alcohol and caffeine consumption, portions of vegetables and fruits, and meat or cold cut intake per day) and urinary sodium and creatinine excretion. Because fluid intake is a major driver of 24UV, we added this variable in a separate step to the multivariable model to explore to what extent the observed differences were attributable to self-reported fluid intake. The relationship between place of living and 24UV was investigated by constructing first a crude model with German-speaking canton as binary variable for living in a German-speaking canton, and second adding stepwise all other covariates to the crude linear regression model as described above. Behavioral and dietary habits were analyzed across linguistic region. To assess differences across gender groups, we used multiplicative sex by covariate interaction tests in the fully adjusted models. Parameter estimates are reported with 95% confidence intervals. We used a two-tailed p value <0.05 to indicate statistical significance. All statistical analyses were performed using SAS version 9.3.

## Results

### Baseline characteristics according to sex

Baseline characteristics stratified by sex are presented in Table 1. Overall, 661 (50.8%) of 1300 participants were female. Median age was not significantly different (men: median [interquartile Range (IQR)] 51 years of age [34, 64] vs women 48 [33, 62]), p = 0.05). Men had a higher BMI (median [IQR] 26 [23, 29] vs 24 [21, 27] kg/m^2^, p < 0.0001), a higher creatinine clearance (median [IQR] 122 [101, 142] vs 96 [79, 114] ml/min, p < 0.0001), a higher prevalence of hypertension (number [percentage] 226 [35%] vs 128 [19%], p < 0.0001), did take more often oral anti-diabetic medication (number [percentage] 30 [5%] vs 14 [2%], p = 0.01), had a higher education level (p < 0.0001), reported a higher alcohol consumption (p < 0.0001), and were more often past smokers (p = 0.0008) than women. Median excreted 24UV in women was 2000 ml (CI: 1354, 2562) compared to 1780 ml (CI: 1244, 2360) in men (p = 0.002) and median urinary sodium excretion was significantly lower in women (128 mmol/24 h [CI: 95, 170] compared to men (176 mmol/24 h [CI: 131, 224], p < 0.0001). Daily fluid intake was similar across sexes (p = 0.86).

**Table 1:** Baseline characteristics according to sex

**Characteristic (n =1300)**	**Women (n = 661)**	**Men (n = 639)**	P value*
Age, years	48 (33 – 62)	51 (34 – 64)	0.05
Fluid intake [Liter/24 h]	1.5 (1.5 – 2.0)	1.5 (1.2 – 2.0)	0.86
Urine volume [Liter/24 h]	2.0 (1.4 – 2.6)	1.8 (1.2 – 2.4)	0.002
Urinary creatinine excretion [mg/kg/24 h]	16.9 (14.0 – 19.8)	21.5 (18.4 – 24.5)	<.0001
Urinary sodium excretion [mmol/24 h]	128 (95 – 170)	176 (131 – 224)	<.0001
Serum creatinine [μmol/L]	70 (63 – 77)	87 (80 – 96)	<.0001
Creatinine clearance [ml/min]	96 (79 – 114)	122 (101 – 142)	<.0001
eGFR (CKD-EPI) [ml/min/1.73 m^2^]	89 (76 – 104)	90 (77 – 103)	0.99
Body mass index [kg/m^2^]	24 (21 – 27)	26 (23 – 29)	<.0001
Hypertension	128 (19%)	226 (35%)	<.0001
Oral anti-diabetic medication	14 (2%)	30 (5%)	0.009
**Education**			<.0001
Primary level	74 (11%)	61 (10%)	
Secondary level	315 (48%)	246 (39%)	
Tertiary level	234 (35%)	302 (47%)	
Other	34 (5%)	29 (5%)	
**Smoking**			0.0008
Current Smokers	106 (16%)	110 (17%)	
Past Smokers	164 (25%)	213 (33%)	
Never Smokers	387 (58%)	306 (48%)	
**Cups of coffee per day**			0.46
0	55 (9%)	42 (7%)	
1-3	443 (67%)	415 (65%)	
≥4	160 (24%)	179 (28%)	
**Alcohol consumption**			<.0001
Never	137 (20%)	53 (8%)	
1 – 2 times/week	402 (61%)	309 (48%)	
> two days/week to daily	99 (15%)	206 (32%)	
twice daily or more	12 (2%)	66 (10%)	
**Vegetables and vegetables juices**			<.0001
< once a day	101 (15%)	183 (29%)	
1 – 2 portions daily	368 (56%)	350 (55%)	
≥3 portions daily	187 (28%)	100 (16%)	
**Fruits and fruit juices**			<.0001
< once a day	126 (19%)	223 (35%)	
1 – 2 portions daily	343 (52%)	310 (49%	
>3 portions daily	189 (29%)	98 (15%)	
**Meat or cold cut**			<.0001
once or < once weekly	142 (21%)	54 (8%)	
2 – 4 days weekly	384 (58%)	318 (50%)	
>5 days weekly	134 (20%)	265 (41%)	

Values are median (interquartile ranges) or counts (percentages). Number of observations across categories may not sum to the given number because of missing data. *eGFR* estimated glomerular filtration rate, *CKD-EPI* Chronic Kidney Disease Epidemiology Collaboration. *Based on Kruskal-Wallis tests for continuous variables and chi-square tests for categorical variables.

### Baseline characteristics according to low versus high 24-hour urine volume

Baseline characteristics stratified by low versus high 24UV are presented in Table 2. Median 24UV was 1.0 L [0.9, 1.2] and 2.9 L [2.7, 3.5] in the lowest and highest quartile of 24UV, respectively. Self-reported fluid intake was significantly lower among low 24UV (low 24UV: 1.5 L [1.0, 1.5] vs high 24UV: 2 [1.5, 2.5]), p < 0.001). Subjects with low 24UV had higher serum creatinine levels and a higher creatinine clearance. The estimated glomerular filtration rate calculated with the Chronic Kidney Disease Epidemiology Collaboration (CKD-EPI) formula was not significantly different between low and high 24UV groups (91 ml/min/1.73 m^2^ [75, 107] vs 93 [81, 105], p = 0.21). Numbers of self-reported history of nephrolithiasis was low in both groups and showed no significant difference (n = 32 [9.7%] vs n = 27 [8.2%], p = 79). Individuals with high 24UV achieved higher education levels (p = 0.01) and had a significantly higher intake of fruits (p = 0.04) and vegetables (p < 0.0001).

**Table 2:** Baseline characteristics according to low versus high 24UV

**Characteristic (n =650)**	**Low 24UV (n =325)**	**High 24UV (n =325)**	P value*
Urine volume [L/24 h]	1.0 (0.9 – 1.2)	2.9 (2.7 – 3.5)	---
Age, years	48 (30 – 63)	47 (35 – 60)	0.97
Fluid intake [L/24 h]	1.5 (1.0 – 1.5)	2.0 (1.5 – 2.5)	<.0001
Urinary creatinine excretion [mg/kg/24 h]	18.5 (15.1 – 22.7)	19.3 (16.1 – 22.5)	0.14
Urinary sodium excretion [mmol/24 h]	120 (94 – 165)	171 (130 – 214)	<.0001
Serum creatinine [μmol/L]	80 (70 – 91)	75 (66 – 86)	0.0002
Creatinine clearance [ml/min]	121 (100 – 142)	109 (90 – 132)	0.0044
eGFR (CKD-EPI) [ml/min/1.73 m2]	91 (75 – 107)	93 (81 – 105)	0.21
Body mass index [kg/m^2^]	24 (22 – 28)	24 (22 – 27)	0.72
Hypertension	82 (25%)	72 (22%)	0.04
Oral anti-diabetic medication	13 (4%)	5 (2%)	0.11
History of nephrolithiasis	32 (9.7%)	27 (8.2%)	0.79
**Education**			0.01
Primary level	44 (14%)	28 (8%)	
Secondary level	152 (47%)	138 (42%)	
Tertiary level	110 (34%)	147 (45%)	
Other	17 (5%)	12 (4%)	
**Smoking**			0.09
Current Smokers	64 (20%)	49 (15%)	
Past Smokers	78 (24%)	99 (30%)	
Never Smokers	178 (55%)	176 (53%)	
**Cups of coffee per day**			0.19
0	27 (8%)	17 (5%)	
1-3	224 (69%)	207 (63%)	
≥4	72 (22%)	100 (31%)	
**Alcohol consumption**			0.10
Never	53 (16%)	39 (12%)	
1 – 2 times/week	172 (53%)	190 (58%)	
> two days/week to daily	71 (22%)	79 (24%)	
twice daily or more	23 (7%)	17 (5%)	
**Vegetables and vegetables juices**			<.0001
< once a day	104 (32%)	53 (16%)	
1 – 2 portions daily	164 (50%)	176 (54%)	
≥3 portions daily	55 (17%)	92 (28%)	
**Fruits and fruit juices**			0.04
< once a day	110 (34%)	68 (21%)	
1 – 2 portions daily	157 (48%	173 (53%)	
>3 portions daily	55 (17%)	82 (25%)	
**Meat or cold cut**			0.08
once or < once weekly	49 (15%)	67 (21%)	
2 – 4 days weekly	187 (58%)	154 (47%)	
>5 days weekly	89 (27%)	103 (32%)	

Urine volume was defined as high and low according to the highest and lowest quartile of 24UV. Values are median (interquartile ranges) or counts (percentages). Number of observations across categories may not sum to the given number because of missing data. *eGFR* estimated glomerular filtration rate, *CKD-EPI* Chronic Kidney Disease Epidemiology Collaboration. *Based on Kruskal-Wallis tests for continuous variables and chi-square tests for categorical variables.

### Factors associated with 24-hour urinary volume

Table 3 explores various factors associated with 24UV, shown with and without taking into account daily fluid intake. In multivariate regression models not accounting for daily fluid intake, urinary sodium excretion and female sex were associated positively with 24UV: β = 4.5 ml (CI: 3.7, 5.4) and 231 ml (CI: 112 – 350), respectively. Creatinine clearance was also positively associated with 24UV (3.4 ml (CI: 0.9, 5.9) higher 24UV per ml/min). Living in a German-speaking canton was associated with a higher 24UV of 307 ml (CI: 202, 411; p < 0.0001). Drinking more than 3 cups of coffee (β = 226 ml [CI: 31, 421]) and vegetable consumption (β = 242 ml [CI: 93, 391] for more than two portions of vegetables daily) were both associated with higher 24UV. Significant negative associations were found for body mass index (β = −31 ml [CI: -46, -15], p = 0.0001), current smoking (β = −216 ml [CI: -349, -81], p = 0.0017), and meat or cold cut consumption (Table 3).

**Table 3:** Factors associated with urine volume [ml/24 h]

**N = 1254**	**Without Fluid Intake in the model**		**With Fluid Intake in the model**	
	**Beta (95% CI)**	**P value**	**Beta (95% CI)**	**P value**
Fluid intake [Liter/day]	---	---	604.4 (539.3, 670.0)	<.0001
Age [years]	2.4 (−1.1, 5.9)	0.18	6.6 (3.4, 9.7)	<.0001
Female sex	230.8 (111.5, 350.0)	0.0002	280.4 (174.4, 386.4)	<.0001
Body mass index [kg/m^2^]	−30.6 (−46.1, -15.4)	0.0001	−31.7 (−45.4, -18.0)	<.0001
German-speaking cantons	306.6 (201.8, 411.4)	<.0001	124.0 (28.9, 219.0)	0.011
Diabetes medication	−18.8 (−280.7, 243.4)	0.89	34.8 (−197.8, 267.4)	0.77
Hypertension	−67.1 (−193.6, 59.4)	0.30	−51.4 (−163.6, 60.9)	0.37
Urinary creatinine excretion [mg/kg/24 h]	−24.9 (−42.7, -7.2)	0.0059	−16.0 (−31.8, -0.2)	0.047
Urinary sodium excretion [mmol/24 h]	4.5 (3.7, 5.4)	<.0001	4.2 (3.4, 4.9)	<.0001
Creatinine Clearance [ml/min]	3.4 (0.9, 5.9)	0.0083	2.4 (0.2, 4.6)	0.036
Current Smoking	−215.5 (−349.2, -81.0)	0.0017	−145.6 (−265.2, -26.1)	0.017
Past Smoking	7.8 (−100.7, 116.3)	0.89	5.9 (−90.4, 102.1)	0.91
**Season**^†^				
Spring	100.7 (−34.3, 235.8)	0.14	40.1 (−79.9, 160.1)	0.51
Summer	−20.4 (−149.5, 108.7)	0.76	−40.1 (−154.7, 74.4)	0.49
Winter	−93.3 (−240.0, 53.4)	0.21	−82.8 (−213.0, 47.4)	0.21
**Education**^†^				
Secondary level	−37.7 (−199.8, 124.3)	0.65	−5.1 (−149.0, 138.7)	0.94
Tertiary level	17.6 (−146.7, 181.9)	0.83	35.0 (−110.8, 180.8)	0.64
Other	−23.8 (−282.3, 234.7)	0.86	−24.5 (−253.9, 204.9)	0.84
**Cups of coffee per day**^†^				
1-3	120.4 (−57.1, 298.0)	0.18	112.0 (−45.6, 269.4)	0.16
≥4	226.2 (31.3, 421.1)	0.023	158.7 (−14.3, 331.8)	0.07
**Alcohol consumption**^†^				
1 – 2 times/week	181.9 (43.5, 320.2)	0.010	173.7 (51.0, 296.5)	0.006
> two days/week to daily	189.3 (27.1, 351.4)	0.022	188.1 (44.2, 332.0)	0.010
twice daily or more	286.5 (51.0, 522.0)	0.017	338.5 (129.5, 547.6)	0.002
**Vegetables**^**†**^				
1 – 2 portions daily	109.9 (−10.4, 230.1)	0.073	89.7 (−17.0, 196.4)	0.09
≥3 portions daily	242.1 (93.1, 391.2)	0.002	156.1 (23.5, 288.8)	0.02
**Fruits and fruit juices**^†^				
1 – 2 portions daily	57.6 (−56.6, 171.8)	0.32	6.6 (−94.9, 108.0)	0.90
>3 portions daily	133.2 (−7.9, 274.2)	0.06	52.0 (−73.4, 177.4)	0.42
**Meat or cold cut**^†^				
2 – 4 days weekly	−184.5 (−323.7, -45.3)	0.009	−163.3 (−286.8, -39.8)	0.01
>5 days weekly	−193.3 (−349.1, -37.5)	0.015	−221.9 (−360.1, -83.6)	0.002

Data are parameter estimates and 95% confidence limits comparing factors associated with24 UV with and without consideration of fluid intake per day. The multivariate model was adjusted for age, sex, body mass index, urine creatinine excretion, urine sodium excretion, hypertension, diabetes, season, living in German- or not German-speaking cantons, education, creatinine clearance, smoking, alcohol and caffeine intake, portions of vegetables and fruits, and meat or cold cut intake per day (n = 1254). ^†^Reference groups as appropriate: Autumn, primary level of education, no caffeine consumption, less than one portion of vegetables and fruits daily, meat or cold cut once or less than once weekly.

Fluid intake was strongly and positively associated with 24UV, as shown in Table 3 (β = 604 ml [539, 670], p < 0.0001). Taking fluid intake into account slightly changed the relationships described above for other covariates, but did not modify the main conclusions (Table 3). Only the association for age became significant (β = 6.6 ml [CI: 3.4, 9.7], p < 0.0001). Alcohol intake remained a significant positive predictor of 24UV. On the other hand, the inverse relationship for urinary creatinine excretion was weakened and the positive association with the amount of daily coffee consumption became non-significant when accounting for fluid intake. Interaction tests suggested that the effect of creatinine clearance on 24UV was stronger among women (p for interaction 0.009), and that the effect of hypertension was stronger among men (p for interaction 0.01). None of the other seven gender by covariate interaction terms was statistically significant.

### Association of linguistic regions with 24-hour urine volume

Table 4 shows the relationships between living in a German-speaking canton and 24UV. These data show that living in the German part of Switzerland was associated with higher 24UV of 377 ml per day (p < 0.0001). While multivariable adjustment for age, sex, BMI, urinary sodium and creatinine excretion and other behavioral and food habits had little effect on this association, additional adjustment for fluid intake substantially attenuated the relationship, but a significant effect of the linguistic region persisted in the fully adjusted model (β = 148 ml [CI: 54, 243], p = 0.0021). We found that eating habits among linguistic regions were significantly different. German speaking subjects consumed significantly less alcohol (p = 0.0009), less vegetables (p < 0.0001) but larger amounts of meat or cold cut (p = 0.005).

**Table 4:** Determinants of 24-hour urine volume according to German-speaking cantons [ml/24 h]

	**Beta**	**95% Confidence Limits**	**P value**
Crude model (n = 1300)	376.8	(280.4, 473.2)	<.0001
Model 1^*^	339.9	(232.8, 447.0)	<.0001
Model 2^†^	306.6	(201.8, 411.4)	<.0001
Model 3^‡^	124.0	(28.9, 219.0)	0.01

Data are parameter estimates and 95% confidence limits comparing German versus non German-speaking regions. ^*^ Adjusted for age, sex, body mass index, hypertension, diabetes medication, education, smoking and season. This model was based on data of 1,290 participants. ^†^ Additional adjustment for creatinine clearance, urinary creatinine and sodium excretion, alcohol and caffeine intake, portions of vegetables and fruits, and meat or cold cut intake per day. This model was based on data of 1,254 participants. ^‡^ Additional adjustment for fluid intake. This model was based on data of 1,254 participants.

## Discussion

In this large, population-based study, we found several independent factors associated with 24UV. The present study adds to the literature by showing that the factors associated with 24UV mainly reflect lifestyle and sociodemographic differences. Since high 24UV has been shown to be associated with conserved kidney function during ageing [3] and reduced risk of CKD in the general population [8], this study may provide some insights into the possible determinants of reduced kidney function. Of note, these population-based findings contrast with those related to patients with CKD, the latter suggesting a possible harm of greater fluid intake [9,10]. High fluid intake alone suppresses vasopressin release [11]. A large randomized study of patients with autosomal polycystic kidney disease showed that tolvaptan, a V2-receptor antagonist, was able to slow down the decline in kidney function [12]. Further, limiting serum vasopressin levels by increasing water intake has been shown to improve kidney function in animal polycystic kidney disease models [13,14]. Current recommendations to preserve kidney function and to prevent the formation of kidney stones used to focus on daily fluid intake [10,15]. However, other determinants have recently been shown to be involved depending on the type of stone [16]. Because the decline of kidney function seems to be slower in those with higher versus lower 24UV [3], we tried to identify associated factors which are possibly determinants of 24UV in two fully adjusted multivariate models. Participants in the highest quintile of fluid intake had 50% lower risk of CKD in two cross-sectional population-based studies [8]. Adding fluid intake as one of the most important determinants for 24UV to the model did change the magnitude of the associations for most other covariates but not the direction of the associations, as shown in Table 3. The median 24UV was in line with prior studies when comparing with previously published population-based data showing a median urine volume of 1.8 L in 2148 participants of a prospective cohort study undertaken in Canada [3]. In the study by Hebert et al. [9], mean urine volume was around 2.5 L in 442 control patients. Average urine volumes ranged between 1.3 and 1.9 L in men and between 1.4 and 2.2 L in women in the study by Peruca et al. [6]. When comparing low versus high 24UV we found no significant difference in self-reported nephrolithiasis. Due to the small number of nephrolithiasis we might not have had enough power to detect a difference.

### Factors negatively associated with 24UV

Negative associations with high 24UV were found for high BMI, current smoking, and high intake of meat or cold cut. As these factors are known to increase the risk of cardiovascular disease [17-19], an independent effect on the age-related renal function decline would be of utmost importance. An increased risk of kidney stone formation has been described for current smokers and might be related to the significantly decreased 24UV in smokers [20]. Meat does contain a lower water content compared to fruits and vegetables in reference to the amount of calories per portion.

### Factors positively associated with 24UV

Age was associated with higher 24UV, possibly related to the decreasing ability of the kidneys to concentrate urine with advancing age [21]. Salt intake and accordingly measured urinary sodium excretion was significantly associated with higher 24UV and might be related to increased thirst. The impact of salt intake on urinary volume in our study is of similar magnitude as that reported by He et al. [22]. However, the association of self-reported fluid intake and 24UV was hardly changed when accounting for urinary sodium excretion. A significant association was found for the intake of vegetables. Drinking alcohol was associated with significantly higher 24UV. This association holds true even in the lowest category of low alcohol consumption and might be related to the reduction in plasma arginine-vasopressin levels [23]. Caffeine intake was found to slightly increase the risk of calcium kidney stones [24] and caffeine is known to have natriuretic and diuretic effects. We found that drinking more than 3 cups of coffee daily significantly increased 24UV but when accounting for fluid intake this association was no longer significant raising the question whether additional water intake or a distinct caffeine effect is the underlying mechanism.

### Sex-specific differences of 24UV

In contrast to a previous meta-analysis on gender differences of 24UV collections [6], we found a significantly larger median 24UV in women compared to men. In multivariate regression models, female sex remained an independent predictor of 24UV. These findings raise the question of whether the increased risk of hypertension and CKD in men is related to lower 24UV. Another benefit of larger 24UV might be a higher voiding frequency and thereby a reduced risk of ascending urinary tract infections. However, women tend to live healthier compared to men in regard to smoking, alcohol consumption and dietary habits with higher intake of fruits and vegetables and lower portions of meat despite a significantly lower level of education [25]. Comorbidities like hypertension, diabetes, and overweight were more often present in men, at least before women reach the menopause.

### Regional differences of 24UV

Living in a German-speaking canton was associated with significantly higher 24UV. Nearly half of this difference could be attributed to self-reported fluid intake. Despite showing significantly different dietary habits taking into account for these played a minor role of this association. It is interesting to see that even within a very narrow geographic region, globally high socio-economic level and homogeneous ethnic spectrum, small cultural differences across different linguistic regions of a single country are associated with significant differences in 24UV that are not entirely explained by fluid intake or other important covariates available in this study.

### Strength and limitations

To our knowledge, this is the first large population-based study to conduct a thorough investigation of factors associated with 24UV. The following limitations have to be taken into account in the interpretation of this study. First, this was a cross-sectional study, which precludes inference of causality. Second, the low participation rate limits the external validity of our findings [7]. The low participation rate in this study may be explained by the unattractiveness of 24-hour urine collection and the two-stage sampling strategy, which implies that the person contacted by phone was not automatically the one randomly selected to enter the study. It is however unlikely that the observed relationships would be substantially different in non-participants. Dietary information, including self-reported fluid intake, was not collected via a standardized and detailed food-frequency questionnaire, which may result in incomplete and/or imprecise dietary information. Our study can be viewed as hypothesis-generating and the observed results should be confirmed in further population-based studies.

## Conclusions

In conclusion, the present study highlights several socio-cultural and nutritional factors as being independently associated with 24UV in the Swiss population. We found intriguing differences in 24UV across linguistic regions within a single geographically confined country, explained only partly by differences in self-reported fluid and alcohol intakes. These data may help fostering additional research on mechanisms involved in the decline in renal function and kidney stone formation.

## Competing interests

The authors declare that they have no competing interests.

## Authors’ contributions

All authors were involved in study conduct. TS performed the statistical analysis and drafted the manuscript. All authors read and approved the final manuscript.

## Collaborators’ information

Additional Swiss Survey on Salt investigators: Binet I., Erne P., Gabutti L., Gallino A., Greminger P., Guessous I., Hayoz D., Meier P., Muggli T., Péchère-Bertschi A., and Suter P.M..

## Pre-publication history

The pre-publication history for this paper can be accessed here:

http://www.biomedcentral.com/1471-2369/14/246/prepub
